# Progress of Hydropathy in Germany

**Published:** 1842-04-30

**Authors:** 


### THE MEDICAL EXAMINER.

### PHILADELPHIA, APRIL 30, 1842.

**Hydropathy or Hydrodusopathy**, the system of curing all diseases by sweating and cold water, continues to flourish in Germany, where it is superseding the infinitesmal humbug, homœopathy. Our readers will find an account of hydropathy, and of the establishment of the water-doctor, Priessnitz, at Graefenberg, in Silesia, in the 33d number of our 3d volume (p. 521.) A manual of the system, lately published by a Mr. Claridge, in England, has elicited the comments of the London medical press, which very severely and justly condemns the indiscriminate application of so violent a mode of treatment to all diseases. This universal sweating of patients, whether labouring under acute or chronic affections, followed by a deluge of cold water externally and internally—the substance of the hydropathic treatment—must, it is obvious, be, in very many cases, a highly dangerous and often a fatal procedure. It is equally probable, however, that in a few chronic diseases it may effect a favorable impression upon the constitution, and, hence, we are not surprised that Priessnitz has established a considerable reputation as a quack, has had during the year 1841, "an archduchess, princes, princesses, counts, barons, &c., &c., in all about five hundred, under his care, and has accumulated about £50,000." It is not a little amusing to observe how the systems of quackery oscillate from extreme to extreme—from the theoretical absurdity of Perkinism to the practical severity of Thomsonism, and from the inert mysticism of homœopathy to the kill-or-cure violence of this Sangrado of cold water. As the hydropathic delusion turns out to be profitable, we should not be surprised to hear of it in time in our own country.

